# Bulk and Single‐Cell Transcriptome Analyses Unravel Gene Signatures of Mitochondria‐Associated Programmed Cell Death in Diabetic Foot Ulcer

**DOI:** 10.1111/jcmm.70319

**Published:** 2024-12-27

**Authors:** Wenqiang Luo, Ning Li, Jin Liu, Duoyu Li, Yongheng Li, Qing Ma, Chuangxin Lin, Liang Lu, Sipeng Lin

**Affiliations:** ^1^ Department of Orthopedics, The First Affiliated Hospital of USTC, Division of Life Sciences and Medicine University of Science and Technology of China Hefei Anhui P. R. China; ^2^ Department of Orthopedics, Shenshan Medical Center, Sun Yat‐sen Memorial Hospital Sun Yat‐sen University Shanwei Guangdong P. R. China; ^3^ Guangdong Provincial Key Laboratory of Cancer Pathogenesis and Precision Diagnosis and Treatment Shanwei Guangdong P. R. China; ^4^ Department of Orthopedic Surgery Shantou Central Hospital Shantou P. R. China

**Keywords:** BCL2, diabetic foot ulcer, LIPT1, mitochondrial, programmed cell death

## Abstract

Mitochondrial programmed cell death (PCD) plays a critical role in the pathogenesis of diabetic foot ulcers (DFU). In this study, we performed a comprehensive transcriptome analysis to identify potential hub genes and key cell types associated with PCD and mitochondria in DFU. Using intersection analysis of PCD‐ and mitochondria‐related genes, we identified candidate hub genes through protein‐protein interaction and random forest analysis. At the single‐cell level, key cell types were further validated based on the expression of hub genes. Additionally, we explored the transcription factors (TFs) regulating hub gene expression and the cellular heterogeneity of DFU. Finally, the expression of key hub genes and TFs was validated in clinical specimens. Our results identified BCL2 and LIPT1 as significantly downregulated hub genes in DFU, with Keratinocytes, as the key cell type. Immunohistochemistry confirmed downregulation of BCL2 and LIPT1 in DFU samples (*p* < 0.05). Additionally, TFs CEBPD and IRF1 were significantly upregulated in DFU, as confirmed by real‐time polymerase chain reaction analysis (*p* < 0.05).

## 1 Introduction

Patients with diabetes are prone to foot ulcers, which are difficult to heal, representing a severe complication of diabetes. Approximately 50%–60% of diabetic patients develop diabetic foot ulcers (DFU) [1]. Globally, there are approximately 18.6 million new cases of diabetic foot patients each year [2]. 80% of lower limb amputations in diabetic patients are due to DFU, with a five‐year survival rate decreasing from 70% to 30% after amputation [3]. DFU impose a significant health and economic burden worldwide [4]. The healing process of a normal wound typically involves four stages: haemostasis, inflammation, proliferation and remodeling [5]. However, the majority of DFU remain stuck in the inflammation stage, leading to impaired wound healing [4]. Most scholars attribute this phenomenon to diabetic neuropathy and microvascular complications induced by a hyperglycaemic environment, yet the underlying molecular mechanisms remain complex and incompletely elucidated [3].

Wound healing primarily involves the participation of fibroblasts, neutrophils, macrophages, keratinocytes, endothelial cells and other cell types [6]. Proper proliferation, death and migration of these cells at different times and in specific spatial arrangements are necessary to promote wound healing [6, 7]. Increasing evidence suggests that in diabetic wound healing processes, the programmed cell death (PCD) of these cells is disrupted, leading to interference and disorder in the healing process [8, 9, 10, 11, 12]. PCD regulated by multiple genes, is a process of active cell demise. It plays a crucial role in the normal development and pathological progression of multicellular organisms and is regulated by endogenous and exogenous factors [13]. Mitochondria are organelles responsible for providing cellular energy and are found in the cytoplasm of most eukaryotic cells. Extensive research highlights their important role in the initiation, signalling and execution of cell death in PCD [14]. Non‐lytic PCD includes apoptosis and autophagy. In this type of PCD, cells degrade themselves or are engulfed by other cells without causing cell rupture and surrounding inflammation [15]. Mitochondria play a central role in the apoptosis process. Mitochondrial outer membrane permeabilisation can release a large amount of cytochrome c, triggering apoptosis through the production of apoptotic bodies and the activation of caspases [16]. In addition to being substrates for autophagy themselves, mitochondria also participate in the formation of autophagosome membranes, regulating autophagic flux [17]. During cellular stress, the health status of mitochondria also influences whether cells undergo autophagy or apoptosis [17]. Pyroptosis is a form of membrane‐destructive cell death caused by activation of inflammasome sensors [18]. It can cause the release of cellular contents, including pro‐inflammatory factors, which are essential to induce and modulate adaptive immunity for the body against pathogens or cell stress [19]. The Gasdermin family are the key proteins in mediating cell pyroptosis [20]. Research shows that the N‐terminal pore‐forming Gasdermin D fragment causes damage and lysis of mitochondrial membranes by binding to cardiolipin and further demonstrated that mitochondrial damage occurs before cell membrane damage [21]. Necroptosis is a lytic PCD accompanied by organelle swelling, nuclear condensation, chromatin digestion and DNA hydrolysis, and is primarily mediated by tumour necrosis factor [22]. There is already substantial evidence indicating a correlation between mitochondrial dysfunction and necrotic apoptosis. RIPK1 mediates mitophagy and promotes necroptosis by enhancing phosphorylation of MLKL, a substrate of RIPK3 [23]. What's more, inflammasomes can elevate mitochondrial ROS, which, through gasdermin D‐mediated membrane permeabilisation, promote necroptosis [24]. In recent years, newly discovered ferroptosis and cuproptosis have also been shown to be closely associated with mitochondria. In ferroptosis, iron and ROS lead to the loss of mitochondrial outer membrane integrity and mitochondrial cristae degradation [25]. Cuproptosis is a form of cell death characterised by the accumulation of copper, leading to the aggregation of lipoylated mitochondrial proteins and instability of Fe‐S cluster proteins [26].

However, in the screening and verifying key biomarkers of mitochondrial‐related PCD in diabetic wounds, analytical bias, accidental bias, screening bias and publication bias would affect the accuracy and reproducibility of the results [27]. Therefore, it is necessary to analyse the transcriptome database of PCD‐related genes. As far as we know, there are currently no published studies on this topic. In addition, different cell PCD modes and effects on wounds in diabetic wounds are different [28, 29, 30, 31, 32, 33]. Therefore, this study integrates single‐cell RNA sequencing (scRNA‐seq) data to deepen our understanding of PCD in diabetic ulcers at the cellular level, which could offer new therapeutic insights for treating diabetic foot ulcers.

In summary, mitochondrial function plays a unique role in various PCD processes, and the PCD of various cells in the wound affects the healing process of diabetic ulcers. However, the impact of mitochondrial‐related PCD in DFU needs further exploration and clarification. This study aims to explore the mechanisms of mitochondrial‐related PCD in DFU by integrating results from single‐cell RNA‐seq and bulk RNA‐seq analyses and validating them in clinical tissue samples.

## 2 Materials and Methods

### 2.1 Data Source

In this study, both single‐cell and bulk transcriptome data from multiple datasets were analysed. The key features of each dataset, including data type, number of genes and samples, are summarised in Table 1. These datasets were downloaded from the Gene Expression Omnibus (GEO) database (https://www.ncbi.nlm.nih.gov/). The datasets of GSE80178 and GSE68183 were merged using the ‘SVA’ package (v3.46.0) as a training set [34]. Additionally, the GSE29221 (GPL6947) as a validation dataset was obtained. A sum of 1548 programmed cell death‐related Genes (PCDRGs) were identified based on the reference [35], while 1136 mitochondrion‐related genes (MRGs) were sourced from the MitoCarta3.0 mitochondrial protein database (http://www.broadinstitute.org/mitocarta) [36].

**TABLE 1:** The key features of each dataset.

Dataset	Platform	Data type	Number of genes	Number of samples	Group information
GSE165816	GPL24676	Single‐cell transcriptome	22,957	21 (14 DFU, 11 NFU)	Foot skin samples from DFU and NFU patients
GSE80178	GPL16686	Bulk transcriptome	16,346	9 (6 DFU, 3 NFU)	Foot skin samples from DFU and NFU patients
GSE68183	GPL16686	Bulk transcriptome	16,346	6 (3 DFU, 3 NFU)	Foot skin samples from DFU and NFU patients
GSE29221	GPL6947	Bulk transcriptome	19,130	6 (3 DM, 3 Control)	Skeletal muscle biopsy samples from type 2 diabetes mellitus (DM) and non‐diabetic (control) male patients

### 2.2 Screening of Candidate Genes for Mitochondrial‐Related PCD in DFU

The differentially expressed genes (DEGs) between DFU and NFU in the training set were selected using the limma package (v3.52.4) with the screening criteria of *p* value < 0.05 & |log_2_FC| > 0.5 [37]. A protein‐protein interaction (PPI) network was constructed based on the String database (http://www.string‐db.org/) to investigate the interactions among the proteins encoded by the intersected genes of the DEGs, MRGs and PCDRGs. Genes demonstrating interactions were designated as candidate genes for subsequent analysis.

### 2.3 Functional Enrichment Analysis of Candidate Genes

To investigate the functional roles and pathways associated with the candidate genes, Gene Ontology (GO) and Kyoto Encyclopedia of Genes and Genomes (KEGG) enrichment analyses were performed using the clusterprofiler package (v3.18.1) with criteria of FDR < 0.05 and *p* < 0.05 [38].

### 2.4 Identification of Candidate Key Cell Clusters by scRNA‐Seq Analysis

#### 2.4.1 Data Preparation for scRNA‐Seq Analysis

Based on GSE165816 single‐cell sequencing data, the data were filtered using the R package Seurat (v5.0.1) [39]. Quality control was performed by filtering out cells with fewer than 200 genes and genes covered by < 3 cells, as well as excluding cells with an expression gene count < 200 and > 7500. Genes with counts < 500 and greater than over 20,000 were also removed, and the proportion of mitochondrial genes was restricted to below 20%. Following data standardisation using the NormalizeData function, the vst method was applied to extract genes with significantly high coefficients of variation among cells, identifying the top 2000 highly variable genes for subsequent analysis. Significant principal components (PCs) were determined using the JackStrawPlot function.

#### 2.4.2 Cell Population Clustering and Annotation

Post‐principal component analysis (PCA) dimensionality reduction, unsupervised clustering analysis was performed on cells using the FindNeighbors and FindClusters functions of the Seurat package (v 5.0.1) to assign cells to distinct clusters, and visualisation was carried out using Uniform Manifold Approximation and Projection (UMAP) [39]. Based on the cell clustering results, the clusters were annotated with marker genes from references [40, 41].

#### 2.4.3 Identification of Candidate Key Cell Clusters

To obtain the key cell clusters of mitochondrial‐related PCD in DFU, GSVA was performed using the GSVA software package (v1.44.5) based on the candidate genes obtained in 2.2 [42]. The ssGSEA scores of each cell type in the DFU and NFU groups were calculated, and the differences in ssGSEA scores of each cell type in the DFU and NFU groups were compared. The cell clusters with *p* < 0.05 were selected as the candidate key cell clusters for this study.

### 2.5 Acquisition of Hub Genes

In the training set, random forest (RF) analysis was conducted based on the candidate genes using the randomForest package (v4.7‐1.1) [43]. The top 5 candidate genes were selected based on the MeanDecreaseGini importance ranking (Seed = 234 and ntree = 1000). Subsequently, in both the training and validation datasets, the expression levels of the candidate genes in DFU and NFU samples were compared and analysed. Genes showing consistent expression trends in both the training and validation sets with significant differences between groups (*p* < 0.05) were defined as hub genes.

### 2.6 Gene Set Enrichment Analysis (GSEA) of Hub Genes

To gain insights into the biological functions of hub genes involved in the DFU development process, GSEA was conducted. In the training set, Spearman correlation coefficients between each hub gene and all other genes were calculated, followed by sorting based on the correlation coefficients from highest to lowest. Subsequently, GSEA was performed using the ClusterProfiler package (v3.18.1) [38] with the ‘c2.cp.v2023.1.Hs.symbols.gmt’ from the Molecular Signatures Database (MSigDB, http://www.gsea‐msigdb.org/gsea/msigdb/index.jsp) as the reference gene set (*p* < 0.05 and FDR < 0.05).

### 2.7 Construction of Gene–Gene Interaction (GGI) Network

To identify other genes functionally related to the hub genes, GeneMANIA (http://www.genemania.org/) was used to construct a GGI network.

### 2.8 Annotation of Transcription Factors (TFs)

The RcisTarget package (v 1.19.2) was utilised to calculate recovery curves based on the motif ranking of hub genes as gene sets, and significant motifs are selected according to the normalised enrichment score (NES) (NES > 0.3), thereby determining the TFs associated with the hub genes [44].

### 2.9 Identification of Key Cell Clusters

The expression levels of hub genes in candidate key cell clusters obtained in 2.4 were visualised and compared, and cell clusters with significant differences in expression between the DFU and NFU groups were defined as key cells. Furthermore, the key cells were explored for their major biological functions using the ReactomeGSA package (v 1.12.0) to further investigate their biological pathways [45].

### 2.10 Heterogeneity of Key Cell

To delve into the heterogeneity of key cells, the FindNeighbors and FindClusters functions from the Seurat package (v5.0.1) were utilised to assign key cell types to different cell clusters based on marker genes, further annotating them into distinct cell subpopulations [39]. Subsequently, the expression of hub genes in different cell subpopulations was compared between the NFU and DFU groups.

### 2.11 Construction of TFs‐mRNA Network

The raw UMI data of key cell types were inputted for single‐cell regulatory network inference and clustering (SCENIC) analysis. Based on AUC > 0 filtering, TFs enriched for hub genes in key cell types were obtained. Simultaneously, the present TFs and the TFs obtained from 2.8 were intersected, resulting in key TFs. A TFs‐mRNA network was constructed using these TFs and hub genes.

### 2.12 Cell Communication Analysis

The proportion and distribution of key cell subpopulations and cell types in the disease and control groups were analysed. Communication analysis between key cell subpopulations and other cell types was conducted using the CellChat package (v1.6.1) [46].

### 2.13 Construction of Molecular Regulatory Network

The miRNAs regulating the hub genes were predicted based on miRDB (https://mirdb.org/) and starbase databases (http://starbase.sysu.edu.cn/index.php). Subsequently, the lncRNAs regulating the predicted miRNAs were identified using the starbase database (http://starbase.sysu.edu.cn/index.php). Finally, the lncRNA‐miRNA‐mRNA network was visualised using Cytoscape software (v3.9.1) [47].

### 2.14 Drug and Diseases Prediction and Molecular Docking

The potential drugs acting on the hub genes were predicted using the Drug Gene Interaction Database (DGidb, https://dgidb.org/). The top 10 drugs based on interaction scores were considered as potential drugs. Subsequently, a drug‐genes network was constructed using Cytoscape (v3.9.1) [47]. Molecular docking between the hub genes and these 10 drugs was performed on the CB‐Dock online platform to validate the binding level. The results were visualised using PyMOL software. Furthermore, the hub genes were input into the Comparative Toxicogenomics Database (CTD, https://ctdbase.org/) to predict potential related diseases.

### 2.15 Clinical Specimen Collection

DFU skin tissues were obtained from 6 patients (3 men and 3 women, averagely aged 60.4 ± 3.0 years) with diabetic foot requiring debridement surgery. Non‐diabetic (healthy) foot skin tissues were collected from 6 patients (2 men and 4 women, averagely aged 62.6 ± 2.6 years) who required open surgery for foot fractures as a natural control (NC) group. This study was approved by the Ethics Committee of Anhui Provincial Hospital (approval number: 2024‐KY‐287) and carried out in accordance with the Declaration of Helsinki. All experiments were conducted with the patients' informed consent.

### 2.16 Haematoxylin and Eosin (HE) and Immunohistochemistry (IHC) Assay

Tissues were promptly fixed in 4% paraformaldehyde for 24 h and subsequently embedded in paraffin. Skin tissue sections were processed by heating, deparaffinizing, rehydrating and sealing. HE staining was performed using commercially available kits according to the manufacturer's instructions (C0105; Beyotime Biotechnology). For IHC, 3 μm sections were heated, deparaffinised and rehydrated. Permeabilisation was done with 0.1% Triton X‐100, and antigen retrieval was achieved using pepsin. This was followed by blocking with 1% bovine serum albumin (BSA) for 1 h and incubation with the primary antibody at 37°C for 2 h. Secondary antibodies conjugated with horseradish peroxidase (HRP) (Goat anti Rabbit, ab6721; Abcam) were then incubated at 37°C for 30 min. Sections were subsequently stained with DAB (P0203; Beyotime Biotechnology) and haematoxylin (Sigma Aldrich), sealed with neutral balsam, and observed under a biomicroscope (DM2000; Leica).

To semi‐quantify the staining results, we employed Bresalier's evaluation system to estimate the average intensity score (IS) [48]. Specifically, two blinded pathologists assessed the sections, randomly selecting ten areas per section to minimise bias. The average IS was based on staining intensity (0 = no staining; 1 = slight staining; 2 = moderate staining; 3 = strong staining) and the corresponding staining scale (F0–F3). The average IS for each section was calculated using the following formula: ∑ (0 × F0 + 1 × F1 + 2 × F2 + 3 × F3).

### 2.17 Real‐Time PCR Analysis

Total RNA was extracted from cultured cells using the RNAiso Plus kit (9109; TaKaRa Biotechnology), and RNA concentrations were measured with a NanoDrop (ND‐2000; Thermo Fisher Scientific). Reverse transcription was performed with the PrimeScript RT Master Mix, followed by real‐time PCR using the UNICON qPCR SYBR Green Master Mix on a LightCycler 96 Real‐Time PCR System (Roche Molecular Systems Inc.). GAPDH was used as a reference for lncRNA and protein‐coding genes, while U6 was used for miRNA. Gene expression was quantified using the $2^{-\Delta \Delta \mathrm{Ct}}$ method.

### 2.18 Statistical Analysis

All bioinformatic analyses were performed within the R environment (version 4.2.2). Data are presented as mean ± SEM unless otherwise specified. Statistical significance was evaluated with the Wilcoxon rank‐sum test in R or the unpaired Student's *t* test using GraphPad Prism 8.0. Significant differences were identified at a significance threshold of *p* value < 0.05. Unless otherwise specified, the reported *p*‐values are unadjusted *p*‐values.

## 3 Results

### 3.1 A Sum of 10 Candidate Genes Were Obtained in the Training Dataset

**FIGURE 1:**
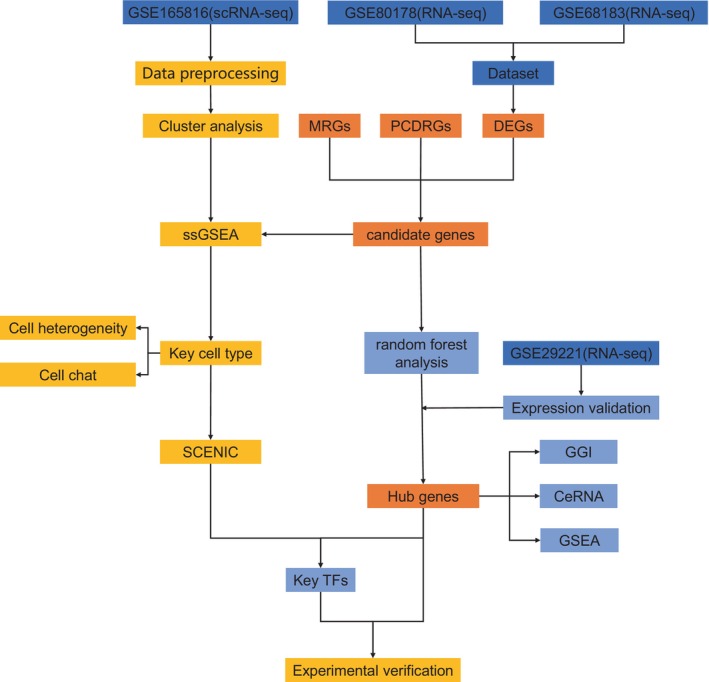
Research design flow chart.

**FIGURE 2:**
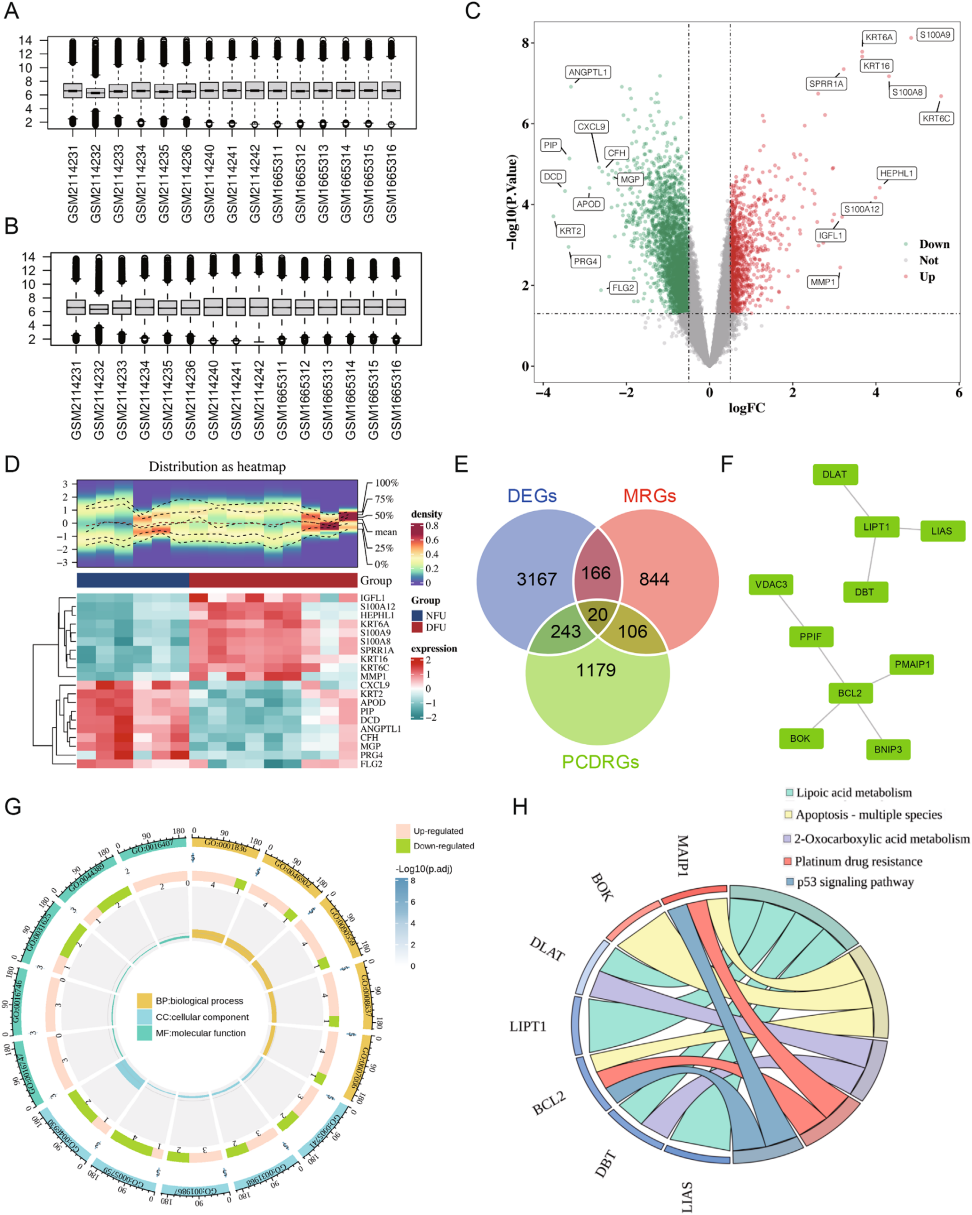
10 candidate genes were obtained in the training dataset. (A, B) The mRNA data from the GSE80178 and GSE68183 datasets were combined (*n* = 9 in the DFU group, *n* = 6 in the NFU group). (C, D) 3596 DEGs were identified in the training set, with 1009 genes upregulated and 2587 genes downregulated. (E) The intersection of DEGs, MRGs and PCDRGs yielded 20 intersecting genes. (F) The PPI network showed that 10 genes were identified to exhibit strong interactions. (G) GO analyses of candidate genes. (H) KEGG analyses of candidate genes.

The research flowchart for this study is presented in Figure 1. The mRNA data from the GSE80178 and GSE68183 datasets were combined to increase the sample size and mitigate batch effects for the training set of this analysis, consisting of 9 DFU and 6 NFU samples (Figure 2A,B). A sum of 3596 DEGs were identified in the training set, with 1009 genes upregulated and 2587 genes downregulated (Figure 2C,D). Subsequently, the intersection of DEGs, MRGs and PCDRGs yielded 20 intersecting genes (Figure 2E). Following this, a PPI analysis was conducted to further explore the interactions between the proteins encoded by these intersecting genes. Within the PPI network, 10 genes were identified to exhibit strong interactions (BCL2, DBT, LIAS, PPIF, BOK, PMAIP1, BNIP3, LIPT1, VDAC3 and DLAT), thus defined as candidate genes for subsequent analysis (Figure 2F). Additionally, GO and KEGG analyses were performed to further elucidate the pathways and functions in which these candidate genes may be involved. Enrichment analysis identified a total of 424 GO items, including examples such as the release of cytochrome c from mitochondria, mitochondrial outer membrane and acyltransferase activity (Figure 2G, Table S1). Furthermore, these candidate genes were enriched in pathways such as the p53 signalling pathway (Figure 2H, Table S2).

### 3.2 Cluster Analysis Revealed 22 Cell Clusters

**FIGURE 3:**
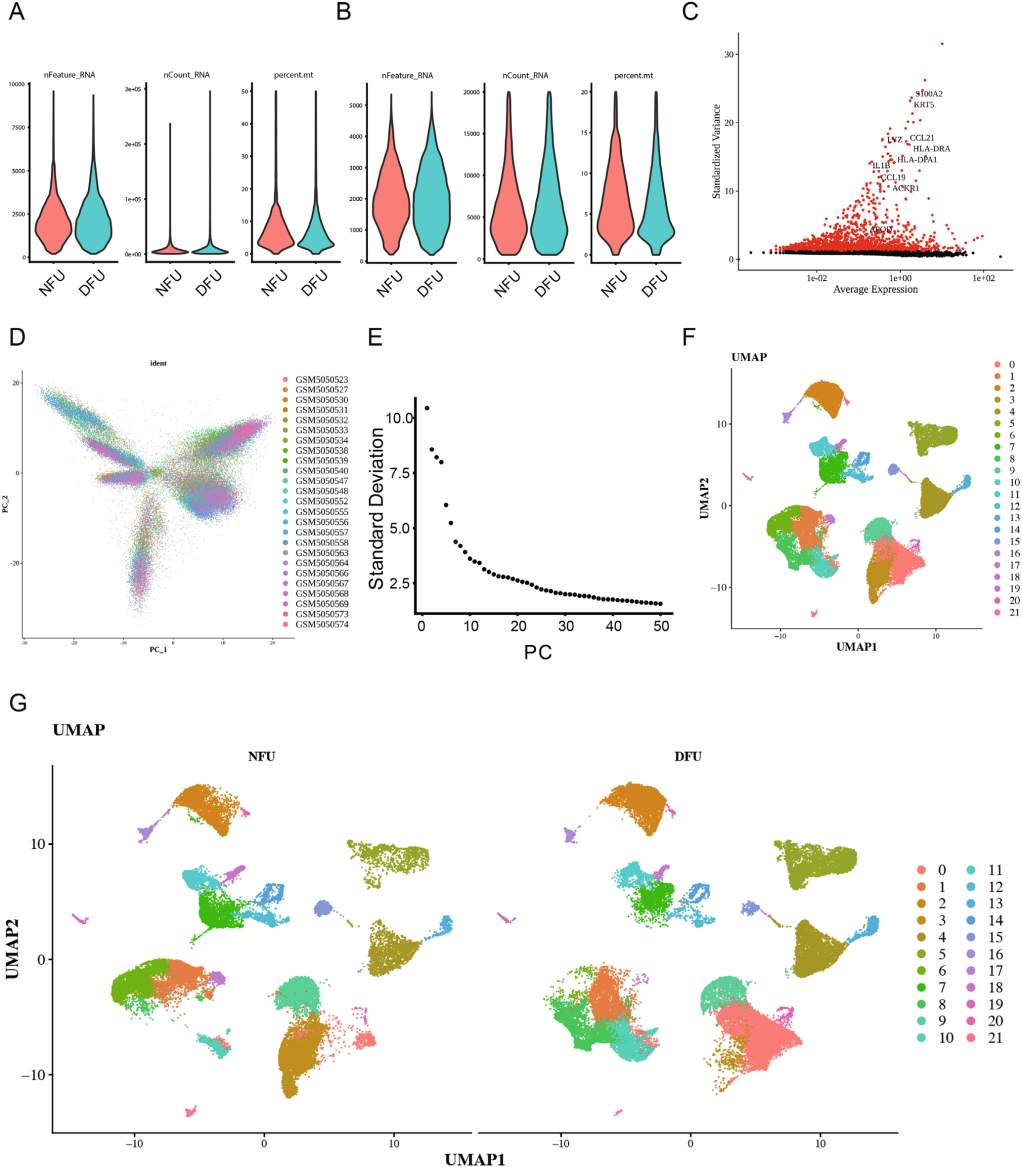
Cluster analysis revealed 22 cell clusters. (A, B) 70,724 cells and 22,957 genes were retained from the single‐cell dataset GSE165816. (C) 2000 highly variable genes were selected for subsequent analysis. (D, E) PCA was conducted using the top 30 PCs for cell clustering and annotation. (F) The results revealed a sum of 22 cell clusters. (G) The NFU (*n* = 11) and DFU (*n* = 14) groups have similar cell clustering patterns.

After filtering, a sum of 70,724 cells and 22,957 genes were retained from the single‐cell dataset GSE165816 (Figure 3A,B). Following data normalisation, 2000 highly variable genes were selected for subsequent analysis (Figure 3C). PCA was further conducted using the top 30 PCs for cell clustering and annotation (Figure 3D,E). The results revealed a sum of 22 cell clusters, with similar cell clustering patterns observed between the NFU and DFU groups (Figure 3F,G).

### 3.3 Kera and Schwann Cells Were Screened as Candidate Key Cell Type

**FIGURE 4:**
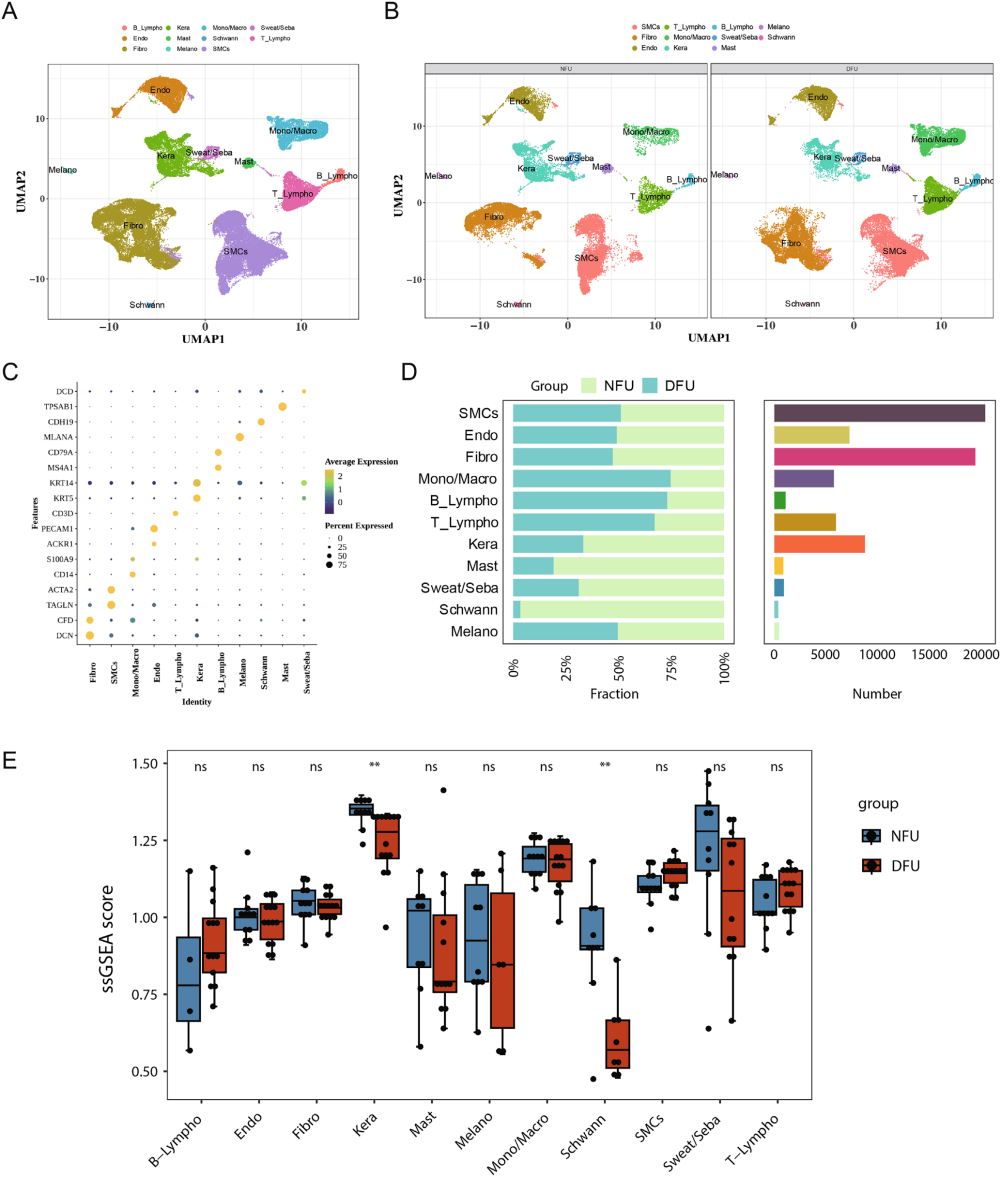
Kera and Schwann cells were screened as candidate key cell types. (A, B) 11 cell types were annotated based on marker genes. (C) Each cell type exhibited high levels of specificity in the expression of marker genes. (D) The fraction and number of each cell type in the DFU (*n* = 14) and NFU (*n* = 11) groups. (E) Differential analysis revealed significant differences in ssGSEA scores between the NFU and DFU groups for Kera and Schwann cells. *p* values were determined using the Wilcoxon rank sum test.

Subsequently, 11 cell types were annotated based on marker genes (Table S3), including B‐lymphocytes (B‐Lympho), keratinocytes (Kera), monocytes/macrophages (Mono/Macro), sweat and sebaceous gland cells (Sweat/Seba), vascular endothelial cells (Endo), mast cells (Mast), Schwann cells (Schwann), T‐lymphocytes (T‐Lympho), fibroblasts (Fibro), melanocytes (Melano) and smooth muscle cells (SMCs) (Figure 4A,B). Furthermore, it was observed that each cell type exhibited high levels of specificity in the expression of marker genes (Figure 4C). Additionally, the proportions of various cell types in the DFU and NFU groups were further statistically analysed, with SMCs being the most abundant cell type in the samples (Figure 4D). Moreover, based on the 20 intersecting genes obtained above, ssGSEA scores were calculated for different cell types between the NFU and DFU groups. Differential analysis revealed significant differences in ssGSEA scores between the NFU and DFU groups for Kera and Schwann cells, with higher scores observed in the NFU group (Figure 4E). Therefore, Kera and Schwann cells were identified as candidate key cells for subsequent analysis.

### 3.4 BCL2 And LIPT1 Were Identified as Hub Genes

**FIGURE 5:**
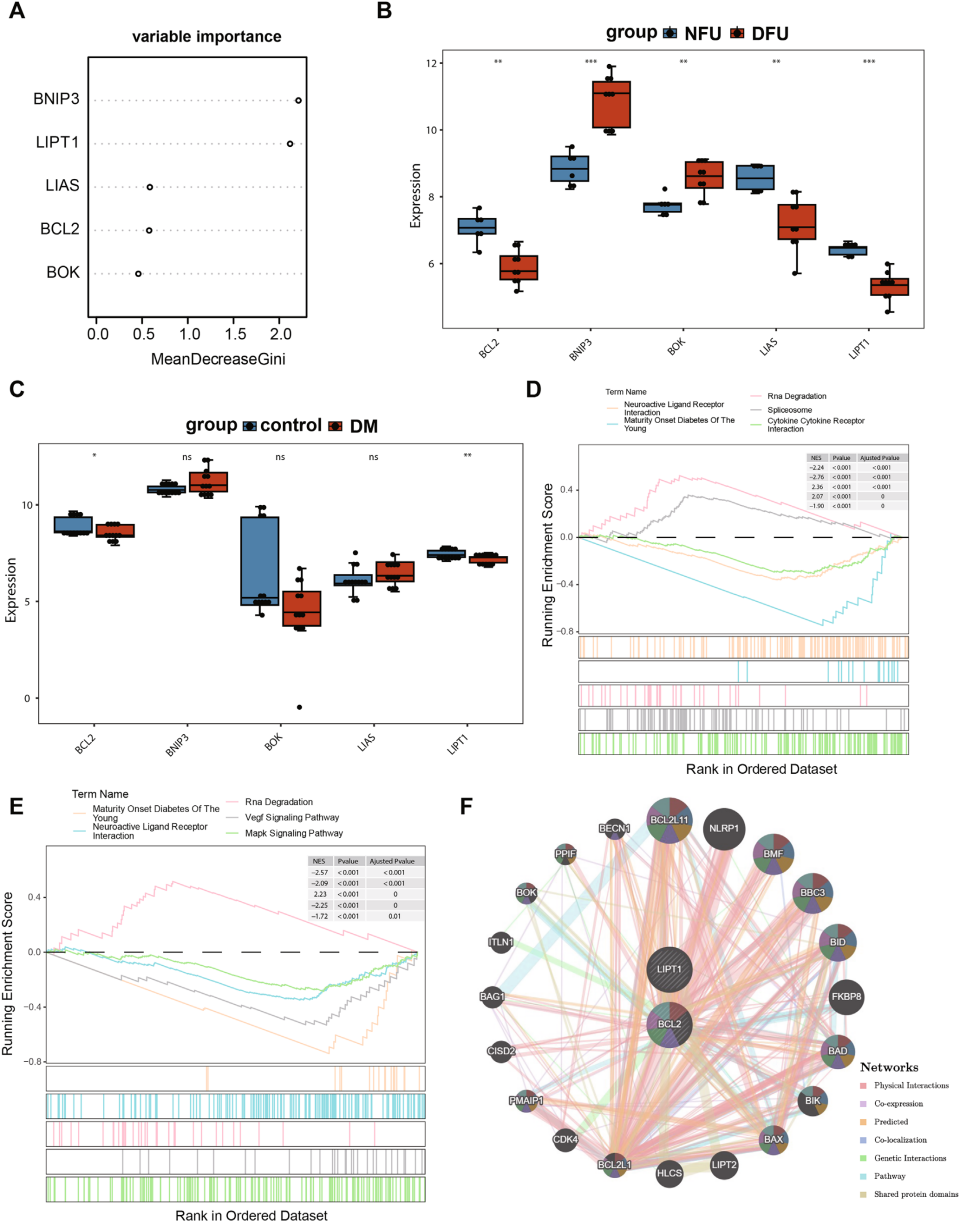
BCL2 and LIPT1 were identified as hub genes. (A) The Top 5 genes were selected based on the ranking of gene importance using MeanDecreaseGini in the RF algorithm. (B, C) BCL2 and LIPT1 exhibited significantly low expression in the DFU (*n* = 9) and DM (*n* = 3) groups. *p* values were determined using the Wilcoxon rank sum test. (D, E) GSEA showed that BCL2 and LIPT1 were enriched in pathways such as neuroactive ligand receptor interaction, maturity onset diabetes of the young and RNA degradation. (F) A GGI network was constructed.

Subsequently, in the RF algorithm, based on the ranking of gene importance using MeanDecreaseGini, the top 5 genes were selected as candidate genes, namely BOX, BCL2, LIAS, LIPT1 and BNIP3 (Figure 5A). Further evaluation of the expression patterns of the candidate genes in both the training set and the GSE29221 dataset revealed that BCL2 and LIPT1 exhibited significantly low expression in the DFU and DM groups, indicating their potential as hub genes for subsequent analysis (Figure 5B,C). Additionally, GSEA was conducted to explore the pathways in which hub genes may be involved. The results indicated that BCL2 and LIPT1 were enriched in pathways such as neuroactive ligand receptor interaction, maturity onset diabetes of the young and RNA degradation (Figure 5D,E). Finally, a GGI network was constructed, revealing that hub genes and their interacting genes were primarily associated with functions related to apoptotic mitochondrial changes, the release of cytochrome c from mitochondria and the regulation of the release of cytochrome c from mitochondria (Figure 5F).

### 3.5 Kera Was Identified as Key Cell Type

**FIGURE 6:**
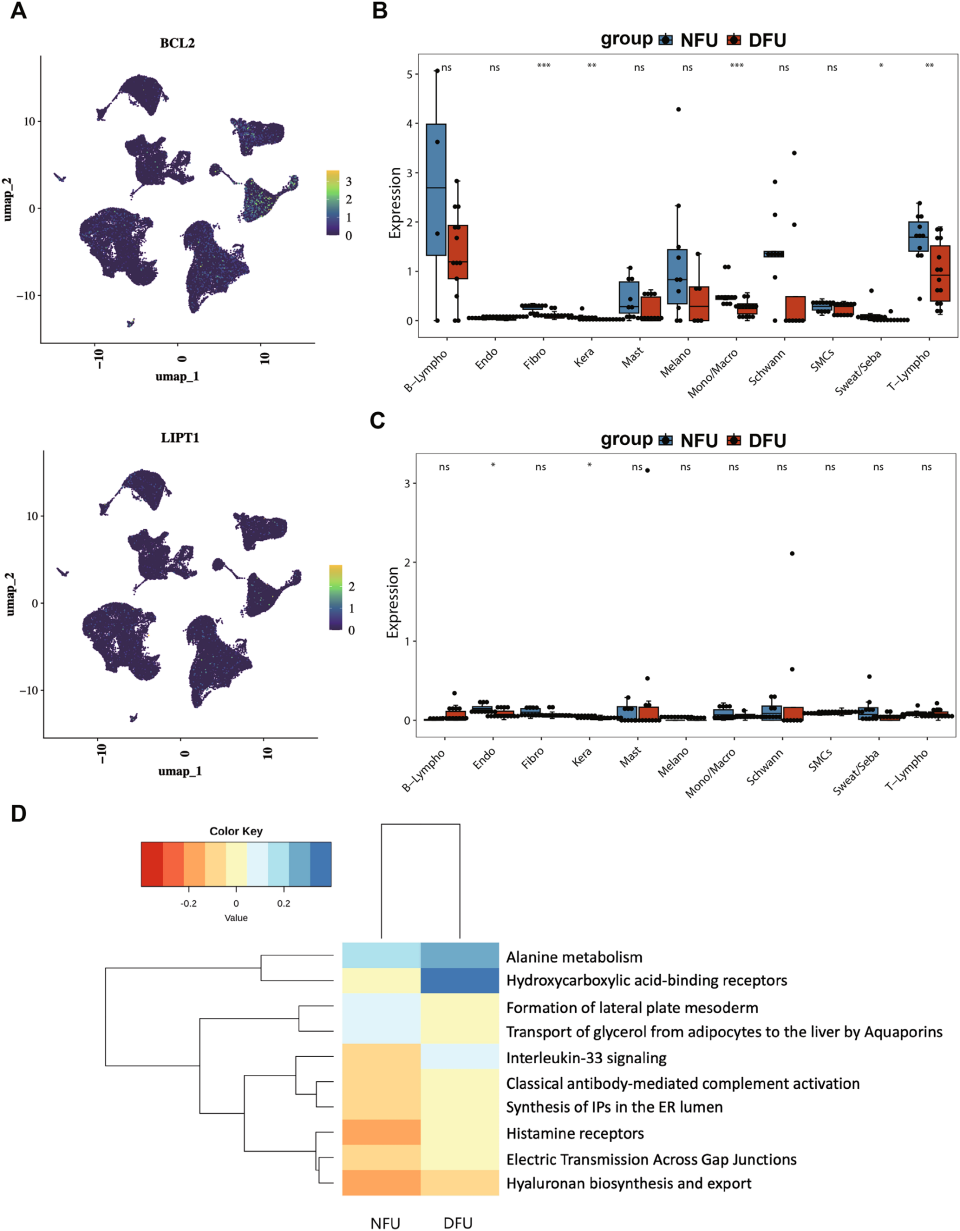
Kera was identified as key cell type. (A) The expression of hub genes in the annotated different cells (*n* = 14 in the DFU group, *n* = 11 in the NFU group). (B, C) The BCL2 gene exhibited significant differences in Fibro, Kera, Mono/Macro, Sweat/Seba and T_Lympho cells and the LIPT1 gene exhibited significant differences in Endo and Kera cells. *p* values were determined using the Wilcoxon rank sum test. (D) The biological functions of Kera were explored.

Further visualisation was performed to show the expression of hub genes in the annotated different cells (Figure 6A). Subsequently, significant differences were observed in the expression of the BCL2 across Fibro, Kera, Mono/Macro, Sweat/Seba and T_Lympho cells, while the LIPT1 gene exhibited significant differences in Endo and Kera cells (Figure 6B,C). By integrating the previously identified candidate key cells, Kera was ultimately defined as the key cell type for subsequent analysis in this study. Finally, enrichment analysis was conducted to further explore the biological pathways in which key cells were involved. The results indicated that the biological functions primarily involved by the Kera include alanine metabolism and hydroxycarboxylic acid receptor binding, etc. (Figure 6D).

### 3.6 The Key Cell Kera Showed Cell Heterogeneity

**FIGURE 7:**
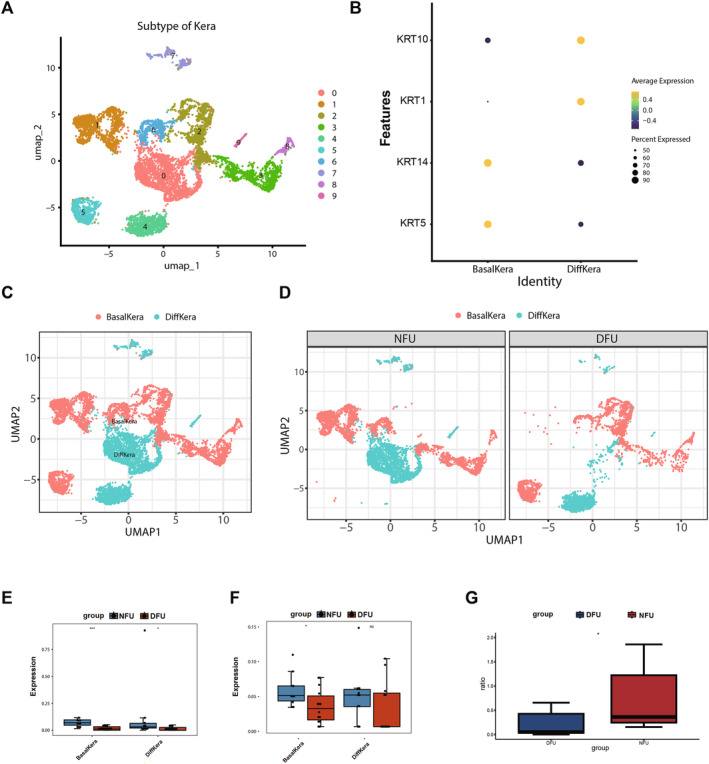
The key cell Kera showed cell heterogeneity. (A) Kera cells could be classified into 10 cell clusters. (B–D) Kera was distinguished into 2 cell subpopulations—BasalKera (KRT5, KRT14) and DiffKera (KRT1, KRT10). (E) The DFU (*n* = 14) group exhibited significant downregulation of BCL2 expression in BasalKera and DiffKera compared to the NFU (*n* = 11) group. (F) LIPT1 expression significantly decreased in BasalKera, but there was no significant difference in DiffKera. (G) The ratio of DiffKera to BasalKera was significantly lower in the DFU group. *p* values were determined using the Wilcoxon rank sum test in (E–G).

To further explore the heterogeneity of key cell types, Kera was subdivided into different cell subpopulations. The results revealed that Kera cells could be classified into 10 cell clusters (Figure 7A). Further subdivision based on marker genes distinguished Kera into 2 cell subpopulations – BasalKera (KRT5, KRT14) and DiffKera (KRT1, KRT10) (Figure 7B,D). To elucidate the expression patterns of hub genes in different cell subtypes, it was observed that compared to the NFU group, the DFU group exhibited significant downregulation of BCL2 expression in BasalKera and DiffKera, while LIPT1 expression was significantly decreased in BasalKera and showed no significant difference in DiffKera (Figure 7E,F). In order to explore whether the diabetic microenvironment has an effect on the differentiation of the two Kera cell subtypes, we performed a ratio analysis of the two cell subtypes and found that the proportion of DiffKera was significantly reduced in the DFU group (Figure 7G).

### 3.7 The Expression of Hub Genes Was Regulated by 6 Key TFs

**FIGURE 8:**
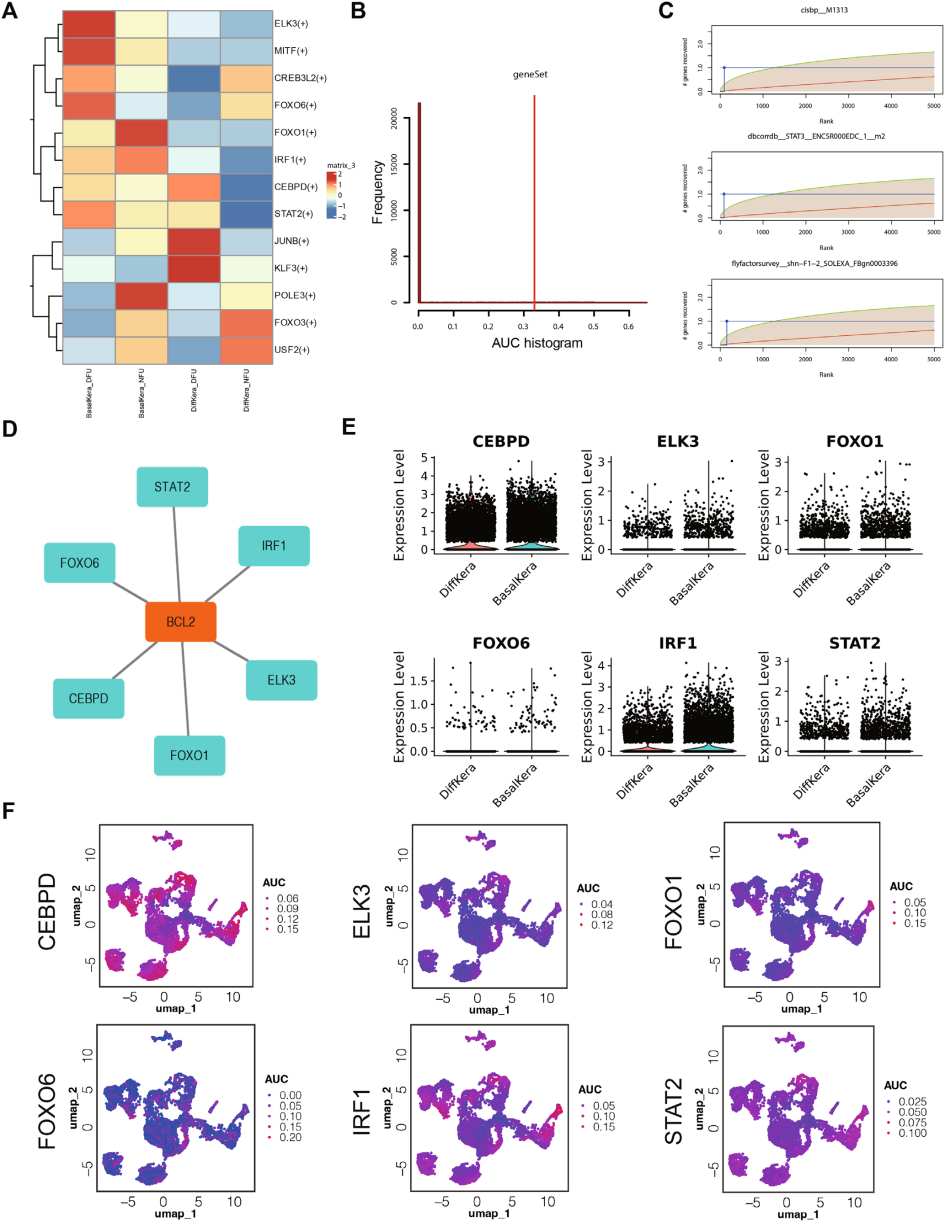
The expression of hub genes was regulated by 6 key TFs. (A) The TFs enriched in the hub genes of the cell subtypes of Kera based on SCENIC analysis (*n* = 14 in the DFU group, *n* = 11 in the NFU group). (B, C) Enrichment of the predicted TFs motifs was assessed using cumulative enrichment curves. (D) The intersection of the obtained TFs resulted in 6 key TFs. (E) Violin plots illustrated the expression levels of the TFs across different cell subtypes. (F) CEBPD and IRF1 exhibited higher AUC values across all cell types on the UMAP dimensions.

The heatmap displayed the TFs enriched in the hub genes of the cell subtypes of Kera based on SCENIC analysis in the NFU and DFU groups (Figure 8A). Furthermore, prediction of the TFs regulating the hub genes was performed using the RcisTarget (Table S4). Initially, enrichment of the predicted TF motifs was assessed using cumulative enrichment curves, and partial motif annotations of some TFs were demonstrated (Figure 8B,C). The intersection of the obtained TFs resulted in 6 key TFs, namely CEBPD, ELK3, FOXO1, FOXO6, IRF1 and STAT2, which were further used to construct a TFs‐mRNA network for visualisation (Figure 8D). Additionally, violin plots illustrated the expression levels of the TFs across different cell types, indicating a higher expression of TFs CEBPD and IRF1 in all cell types (Figure 8E). On the UMAP dimensions, CEBPD and IRF1 exhibited higher AUC values across all cell types, suggesting elevated activity of these two TFs (Figure 8F).

### 3.8 Interactions Between Different Cells

**FIGURE 9:**
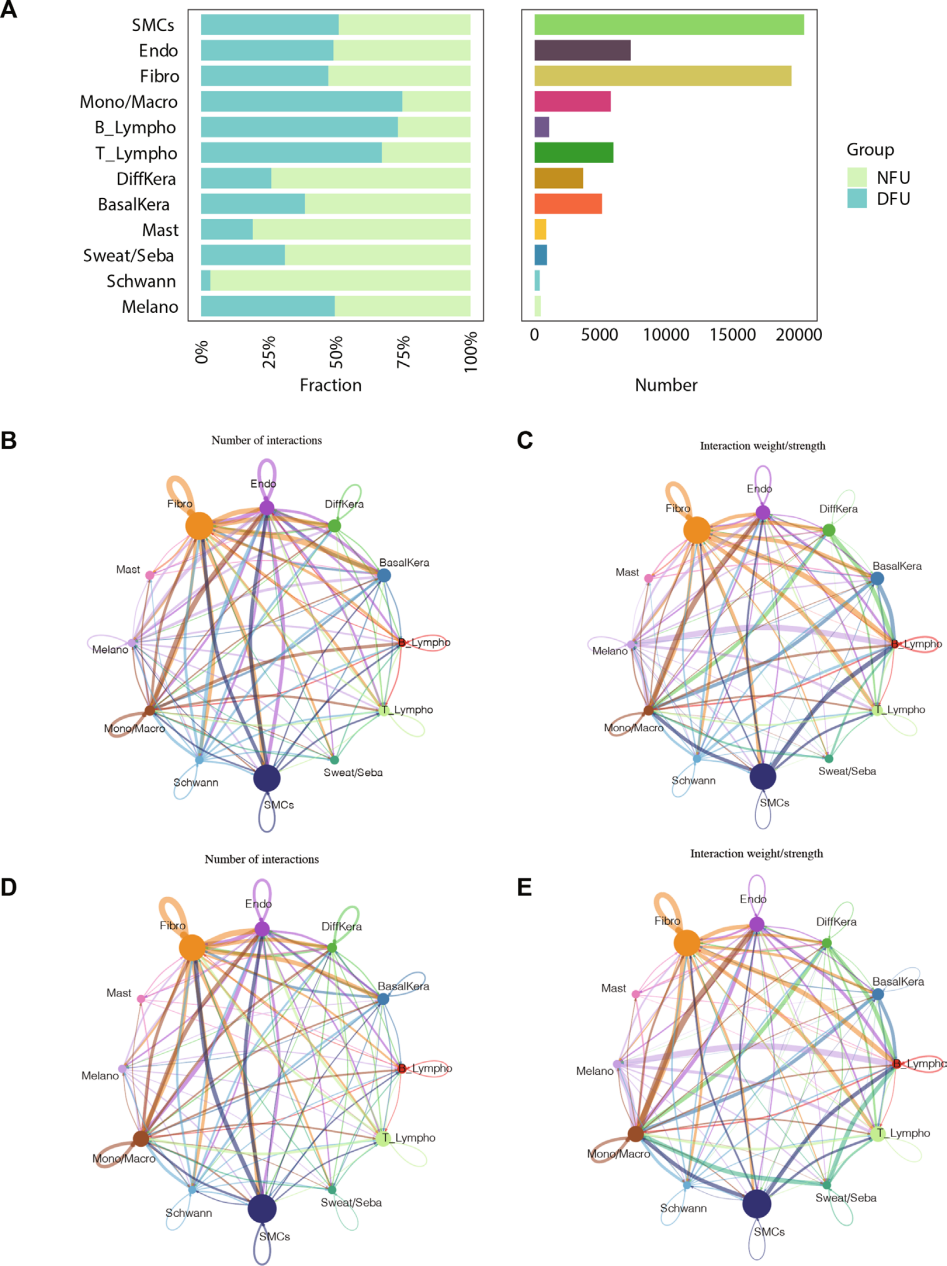
Interactions between different cells. (A) The proportions of Kera cell subpopulations and other cell types were compared between DFU (*n* = 14) and NFU (*n* = 11). In NFU (B, C) and DFU (D, E), Fibro and Endo exhibited a higher number and stronger intensity of interactions with other cell types. Interaction between Diffkera and Sweat/Seba, as well as SMCs in the DFU group, was enhanced.

As shown in Figure 9A, the proportions of Kera cell subpopulations and other cell types were compared between DFU and NFU, with a significant decrease in the proportions of DiffKera and BasalKera in DFU. Subsequently, cell communication was conducted to further explore the interactions between these two cell subpopulations and other cell types. The results revealed that both in NFU and DFU, Fibro and Endo exhibited a higher number and stronger intensity of interactions with other cell types (Figure 9B–E).

### 3.9 Potential Regulatory Network and Drug Prediction

**FIGURE 10:**
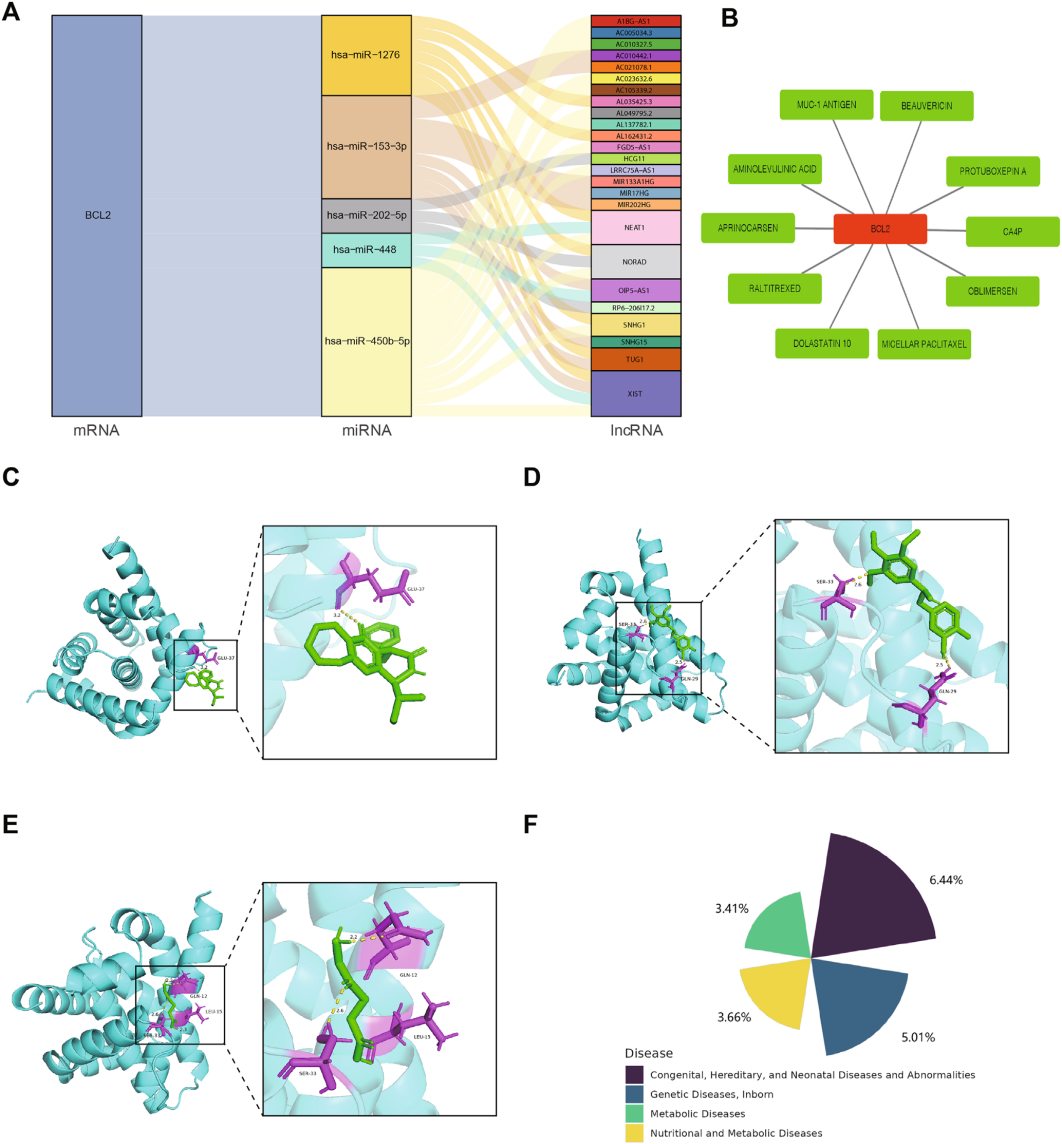
Potential regulatory network and drug prediction. (A) 1 mRNA (BCL2), 5 miRNAs and 25 lncRNAs were ultimately selected for constructing a lncRNA‐miRNA‐mRNA regulatory network. (B) The drug‐genes network constructed included the top 10 drugs based on interaction scores. (C–E) Molecular docking was then used to demonstrate the docking effects of the predicted drugs. (F) The hub genes might be associated with certain metabolic diseases or other diseases.

Based on the database, 1 mRNA (BCL2), 5 miRNAs and 25 lncRNAs were ultimately selected for constructing a lncRNA‐miRNA‐mRNA regulatory network. Relationships such as XIST‐hsa‐miR‐448‐BCL2, AL035425.3‐hsa‐miR‐1276‐BCL2, and others were observed in the network (Figure 10A). Based on the database, drugs potentially interacting with hub genes were further predicted. The drug‐genes network constructed included the top 10 drugs based on interaction scores (Figure 10B). Molecular docking was then used to demonstrate the docking effects of the predicted drugs PROTUBOXEPIN A, CPA4 and AMINOLEVULINIC ACID with BCL2 (Figure 10C–E). Based on the database, it was found that hub genes might be associated with certain metabolic diseases or other diseases such as congenital or hereditary disorders (Figure 10F).

### 3.10 The Expression of BCL2, LIPT1 and TFs Were Verified in Clinical Tissue Specimens

**FIGURE 11:**
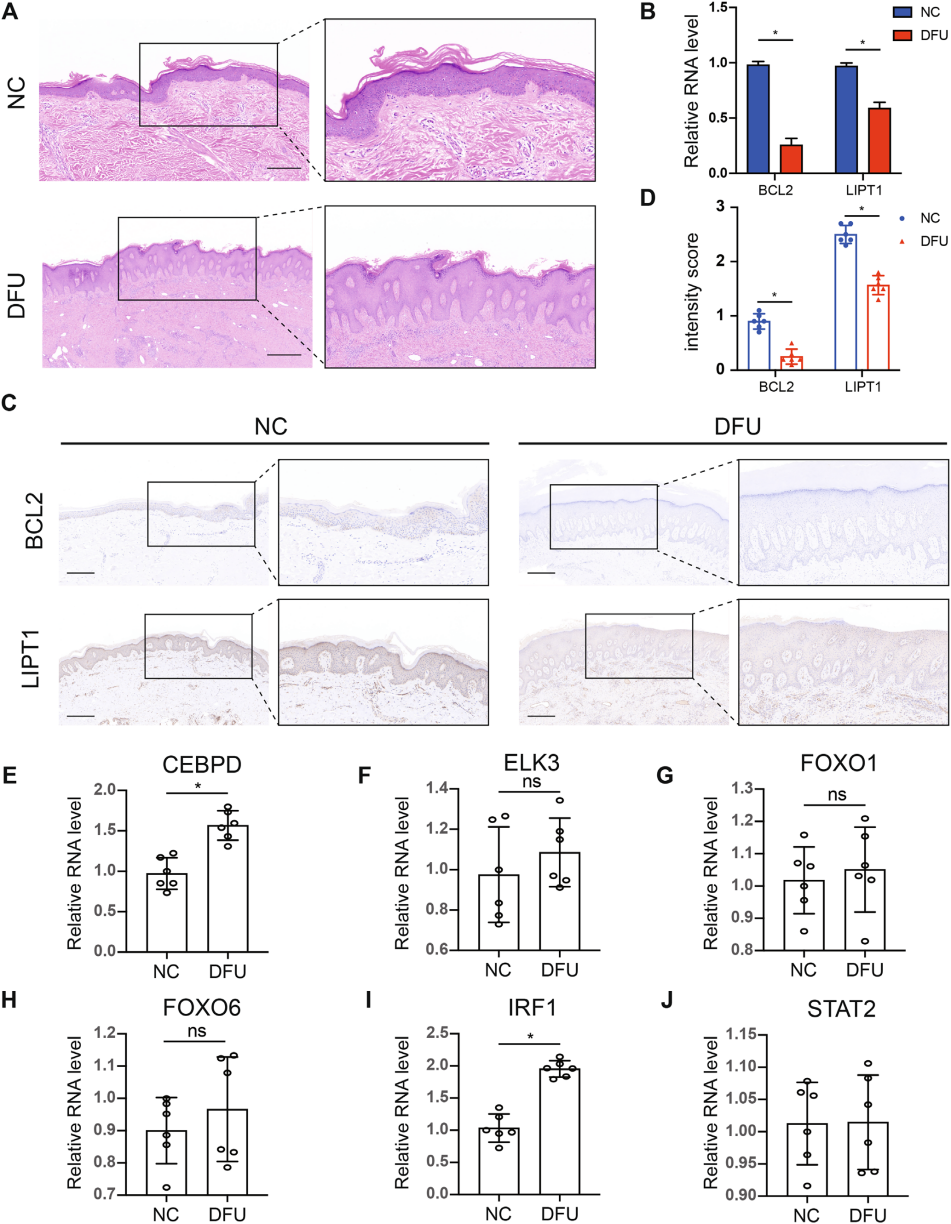
The expression of BCL2, LIPT1 and TFs was verified in clinical tissue specimens. (A) HE staining of the skin tissue of the DFU group (*n* = 6) and the NC (*n* = 6) group. Scale bar, 500 μm. (B) PCR tests showed that BCL2 and LIPT1 were downregulated in the DFU group (*p* < 0.05). (C, D) IHC staining of the DFU group achieved a lower average intensity score (*I*_S_) of BCL2 and LIPT1 (*p* < 0.05). Scale bar, 500 μm. (E–J) The mRNA level of TFs was decreased in the DFU group and NC group (*p* < 0.05). *p < 0.05. A two‐tailed Student's *t*‐test was used for judging statistical significance, with numerical results displayed in the form of mean ± SEM. All experiments were done in sextuplicate.

To further explore the expression of BCL2, LIPT1 and TFs in clinical tissue specimens, we harvested the skin tissue of the DFU group and the NC group. Firstly, HE staining of skin tissues revealed significant alterations in the traumatised skin of patients in the DFU group, characterised by the extension of the epidermis into the dermis and marked thickening of the spinous layer (Figure 11A). To further validate our biochemical analysis, we measured the mRNA expression level of BCL2 and LIPT1 in DFU patients and their controls using RT‐PCR. The results demonstrated that BCL2 and LIPT1 were downregulated in the DFU group (*p* < 0.05) (Figure 11B). Additionally, immunohistochemical analysis showed lower expressions of BCL2 and LIPT1 in the tissues of the DFU group compared with the control group, which was further corroborated by intensity score (Is) (*p* < 0.05) (Figure 11C,D). Subsequently, TFs expression was examined in both groups using RT‐PCR, revealing that CEBPD and IRF1 were significantly elevated in the DFU group, suggesting these factors may serve as upstream regulatory molecules of BCL2 and LIPT1 (*p* < 0.05) (Figure 11E–J).

Bcl‐2 exerts its critical survival function through physical interactions with pro‐apoptotic members of the Bcl‐2 family [49]. Consequently, we analysed the mRNA levels of apoptosis‐related genes Bcl‐2, Bax, Bcl‐xL and Puma in the tissues of both the NC and DFU groups. Apoptosis is regulated by a complex interplay between two groups of Bcl‐2 family members: pro‐apoptotic proteins (such as Bax/Bak‐like proteins and BH3‐only proteins, including Puma, Bad and Bmf) and anti‐apoptotic proteins (such as Bcl‐2, Bcl‐xL and Mcl‐1) [49]. Bcl‐2 can inhibit mitochondrial permeability and subsequent cell death induced by pro‐apoptotic Bax and Bak. This functional inhibition is partly dependent on Bcl‐2's ability to bind the conserved BH3 domain of pro‐apoptotic Bcl‐2 family proteins through its hydrophobic groove [50]. Compared to the control group, DFU tissues exhibited lower expression of anti‐apoptotic proteins Bcl‐2 and Bcl‐xL, along with an upregulation of pro‐apoptotic proteins Bax and Puma (*p* < 0.05) (Figure S1A–D).

## 4 Discussion

To date, some literature has indicated that mitochondrial‐related PCD plays an important role in diabetic wound healing [51, 52, 53, 54]. However, there is currently no systematic bioinformatics study on the molecular mechanisms of mitochondrial‐related PCD in DFU. This study first used bulk RNA‐seq analysis to screen for differentially expressed genes related to mitochondrial PCD in DFU and NFU. Key differentially expressed genes were further selected through PPI and random forest analysis and validated in another bulk RNA‐seq dataset, resulting in the identification of two key genes: BCL2 and LIPT1. Additionally, the differentially expressed genes identified from the bulk RNA‐seq analysis were used as a gene set for GSVA on single‐cell RNA‐seq samples. Candidate key cell clusters, keratinocytes and Schwann cells were discovered. In single‐cell RNA‐seq analysis, the BCL2 gene showed significant differences in Fibro, Kera, Mono/Macro and Sweat/Seba cells, while the LIPT1 gene exhibited significant differences in Endo and Kera cells. Hence, Kera was defined as the key cell cluster. To further validate the main results of the bioinformatics analysis, in vitro experiments were conducted to confirm the expression levels of BCL2, LIPT1 and TFs in DFU and NFU samples, showing consistency with the previous analytical results. This study identifies the hub genes, BCL2 and LIPT1, and key cell cluster, keratinocytes, associated with mitochondrial‐related PCD in DFU, and explores the underlying molecular mechanisms.

BCL2 is a protein located on chromosome 18 that inhibits apoptosis [55]. However, research on BCL2 in DFU is limited. Clinical specimen research by Sivakamasundari et al. also found decreased BCL2 expression in DFU tissues [56]. However, this study did not specify the cell types with reduced BCL2. Our research identifies a significant difference in BCL2 expression specifically in Fibro, Kera, Mono/Macro, Sweat/Seba and T‐Lympho cells. A study on cardiomyocyte‐specific Bmal1 knockout mice demonstrated that Bmal1 can exacerbate diabetic cardiac injury by inhibiting BCL2 transcription, leading to mitochondrial calcium overload and apoptosis [57]. This is consistent with our findings of significant BCL2 downregulation in the DFU group. Similarly, studies have confirmed that kidney biopsies from patients with diabetic nephropathy show significantly reduced BCL2 expression [58]. Wogonin can directly bind to BCL2 and regulate the autophagy and apoptosis of glomerular podocytes, thereby alleviating diabetic nephropathy [58]. In summary, BCL2 is reduced in various target organs affected by diabetes, accelerating apoptosis of the corresponding cells and exacerbating diabetes‐related damage. Diabetic ulcers have difficulty transitioning from the inflammatory phase to the proliferative phase, which may be due to decreased BCL2 in keratinocytes, increased keratinocyte apoptosis and impaired wound re‐epithelialisation.

As a gene associated with cuproptosis, Lipoyltransferase 1 (LIPT1) is an essential enzyme for the mitochondrial 2‐ketoacid dehydrogenase complex involved in lipoylation [59]. It is positively correlated with angiogenesis and cell differentiation and negatively correlated with cell death and DNA damage [60]. Zhimin et al. identified LIPT1 as one of the key genes involved in cuproptosis during diabetic immune infiltration through gene chip data analysis [61]. Similarly, research has found that LIPT1 expression is significantly increased in the synovium of osteoarthritis, making it a potential key gene or therapeutic target for immune infiltration related to cuproptosis [62, 63]. The present study identified LIPT1 as a key gene in mitochondrial‐related PCD in DFU and found that its expression is significantly reduced in keratinocytes and endothelial cells. Based on previous research, we speculate that the immune microenvironment in DFU may lead to abnormal cuproptosis through LIPT1 in keratinocytes and endothelial cells, thereby inhibiting their regeneration.

Previous studies have already identified that various forms of PCD in multiple cell types affect the healing of diabetic wounds [30, 52, 64, 65]. This study, through analysis from both bulk RNA‐seq and single‐cell RNA‐seq data dimensions, suggests that apoptosis or cuproptosis of keratinocytes may be one of the essential reasons for the poor healing of DFU. Ying Liang et al.'s in vitro study found that advanced glycation end products increase matrix metalloproteinase‐9 and induce keratinocyte apoptosis through the FasL/Fas pathway. Although this study also indicates increased keratinocyte apoptosis in diabetic wounds, it does not mention whether this pathway is related to BCL2 [66]. A previous study has shown that a high glucose environment can increase reactive oxygen species production in mitochondria, leading to the induction of IL‐8 production in keratinocytes, and the resulting increased neutrophil infiltration leads to impaired wound healing [67]. Another study found that keratinocytes can transform macrophages into a pro‐healing phenotype and reduce apoptosis through exosomal MALAT1 [68]. These studies indicate that the cellular interactions between immune cells and keratinocytes are closely related to diabetic wound healing. Our study found an enhanced interaction between Diffkera and Sweat/Seba, as well as SMC cells in the DFU group, which has not been reported in previous studies, possibly due to less research on Sweat/Seba and SMC cells in diabetic wounds.

CCAAT/enhancer‐binding protein delta (CEBPD) is expressed at low levels in most normal tissues, upregulated by inflammatory factors, hormones and the tumour microenvironment [69]. Previous studies have indicated that its role in inflammation and tumours is bidirectional; however, in most cases, CEBPD promotes inflammation [70]. This study identified for the first time increased CEBPD in DFU, which may be induced by the hypoxic and chronic inflammatory environment of these wounds [70, 71]. Similarly, Interferon regulatory factor 1 (IRF1) has a low baseline expression level but responds highly to various inflammatory stimuli [72]. Notably, it is primarily expressed in skin keratinocytes rather than immune cells [73]. IRF1 is known to have multiple roles, including resisting infection, inhibiting tumours, suppressing proliferation and promoting apoptosis [74]. The prolonged pro‐inflammatory and infection‐prone environment of DFU undoubtedly leads to increased IRF1 expression. Additionally, this study found that IRF1 may be a key transcription factor involved in keratinocyte apoptosis in DFU, which is consistent with its molecular characteristics and functions.

An obvious shortcoming of our study is the lack of exploration of the specific mechanisms between the key genes, TFs and key cells discovered. Although we have analysed the possible molecular pathways and functional enrichment through extensively utilised bioinformatics analyses and computational methodologies and even made drug predictions, these results need further experimental verification, which is also the direction of our next work.

## 5 Conclusion

The present study, through a combination of bulk RNA‐seq and single‐cell RNA‐seq analysis, along with validation through clinical specimens, identified BCL2 and LIPT in keratinocytes as key therapeutic targets to promote the healing of DFU. This is the first systematic study on the relationship between mitochondrial‐related PCD and diabetic foot, providing new insights for the future treatment of diabetic foot.

## Author Contributions

**Wenqiang Luo:** conceptualization (equal), data curation (equal), writing – original draft (lead). **Ning Li:** conceptualization (equal), data curation (equal), writing – original draft (equal). **Jin Liu:** data curation (equal), formal analysis (equal), software (equal), validation (equal). **Duoyu Li:** data curation (equal), formal analysis (equal), methodology (equal), software (equal). **Yongheng Li:** conceptualization (equal), data curation (equal), formal analysis (equal), methodology (equal). **Qing Ma:** conceptualization (equal), data curation (equal), formal analysis (equal), methodology (equal). **Chuangxin Lin:** writing – review and editing (equal). **Liang Lu:** conceptualization (equal), data curation (equal), supervision (equal), writing – review and editing (equal). **Sipeng Lin:** conceptualization (equal), methodology (equal), supervision (equal), validation (equal), writing – review and editing (lead).

## Conflicts of Interest

The authors declare no conflicts of interest.

## Supporting information


**Figure S1.** The expression of BCL2‐related apoptosis genes in clinical tissue specimens. (A–D) PCR tests showed that Bcl‐2 and Bcl‐xL were downregulated and Bax and Puma were upregulated in the DFU group (*p* < 0.05). *p < 0.05. Two‐tailed Student’s *t*‐test was used for judging statistical significance with numerical results displayed in the form of mean ± SEM. All experiments were done in sextuplicate.

**Table S1.** GO analyses of candidate genes.

**Table S2.** KEGG analyses of candidate genes.

**Table S3.** Marker genes of 11 cell types.

**Table S4.** Predicted TFs of hub genes.

## Data Availability

The processed gene expression data of scRNA‐seq (GSE165816) and bulk RNA‐seq (GSE80178, GSE68183 and GSE29221) are available in the Gene Expression Omnibus (GEO) database (https://www.ncbi.nlm.nih.gov/).

## References

[1] H. Deng , B. Li , Q. Shen , et al., “Mechanisms of Diabetic Foot Ulceration: A Review,” Journal of Diabetes 15, no. 4 (2023): 299–312. 36891783 10.1111/1753-0407.13372 PMC10101842

[2] D. G. Armstrong , T. W. Tan , A. J. M. Boulton , and S. A. Bus , “Diabetic Foot Ulcers: A Review,” Journal of the American Medical Association 330, no. 1 (2023): 62–75. 37395769 10.1001/jama.2023.10578 PMC10723802

[3] F. Huang , X. Lu , Y. Yang , et al., “Microenvironment‐Based Diabetic Foot Ulcer Nanomedicine,” Advanced Science (Weinheim) 10, no. 2 (2023): e2203308. 10.1002/advs.202203308 PMC9839871 36424137

[4] A. Godavarty , K. Leiva , N. Amadi , D. C. Klonoff , and D. G. Armstrong , “Diabetic Foot Ulcer Imaging: An Overview and Future Directions,” Journal of Diabetes Science and Technology 17, no. 6 (2023): 1662–1675. 37594136 10.1177/19322968231187660 PMC10658670

[5] L. Chen , S. Sun , Y. Gao , and X. Ran , “Global Mortality of Diabetic Foot Ulcer: A Systematic Review and Meta‐Analysis of Observational Studies,” Diabetes, Obesity & Metabolism 25, no. 1 (2023): 36–45. 10.1111/dom.14840 36054820

[6] M. Rodrigues , N. Kosaric , C. A. Bonham , and G. C. Gurtner , “Wound Healing: A Cellular Perspective,” Physiological Reviews 99, no. 1 (2019): 665–706. 30475656 10.1152/physrev.00067.2017 PMC6442927

[7] J. Song , K. Zhu , H. Wang , M. Wu , Y. Wu , and Q. Zhang , “Deciphering the Emerging Role of Programmed Cell Death in Diabetic Wound Healing,” International Journal of Biological Sciences 19, no. 15 (2023): 4989–5003. 37781514 10.7150/ijbs.88461 PMC10539695

[8] S. J. Yoon , C. J. Lim , H. J. Chung , et al., “Autophagy Activation by Crepidiastrum Denticulatum Extract Attenuates Environmental Pollutant‐Induced Damage in Dermal Fibroblasts,” International Journal of Molecular Sciences 20, no. 3 (2019): 517. 30691106 10.3390/ijms20030517 PMC6386979

[9] W. Shi , Y. Wu , and D. Bian , “p75NTR Silencing Inhibits Proliferation, Migration, and Extracellular Matrix Deposition of Hypertrophic Scar Fibroblasts by Activating Autophagy Through Inhibiting the PI3K/Akt/mTOR Pathway,” Canadian Journal of Physiology and Pharmacology 99, no. 4 (2021): 349–359. 32726570 10.1139/cjpp-2020-0219

[10] Y. F. Yuan , S. K. Das , and M. Q. Li , “Vitamin D Ameliorates Impaired Wound Healing in Streptozotocin‐Induced Diabetic Mice by Suppressing Endoplasmic Reticulum Stress,” Journal Diabetes Research 2018 (2018): 1757925. 10.1155/2018/1757925 PMC5863297 29707582

[11] L. Huang , H. A. Cai , M. S. Zhang , R. Y. Liao , X. Huang , and F. D. Hu , “Ginsenoside Rg1 Promoted the Wound Healing in Diabetic Foot Ulcers via miR‐489‐3p/Sirt1 Axis,” Journal of Pharmacological Sciences 147, no. 3 (2021): 271–283. 34507636 10.1016/j.jphs.2021.07.008

[12] N. Meng , X. Mu , Y. Gong , et al., “Autophagy Induced by a Novel Triazol Derivative Promotes Angiogenesis Through Decreasing Interferon‐Inducible Protein 10 Level in Vascular Endothelial Cells,” Journal of Cardiovascular Pharmacology 78, no. 1 (2021): e136–e146. 34009854 10.1097/FJC.0000000000001034

[13] M. Kulkarni and J. M. Hardwick , “Programmed Cell Death in Unicellular Versus Multicellular Organisms,” Annual Review of Genetics 57, no. 1 (2023): 435–459. 10.1146/annurev-genet-033123-095833 PMC11491101 37722687

[14] T. T. Nguyen , S. Wei , T. H. Nguyen , et al., “Mitochondria‐Associated Programmed Cell Death as a Therapeutic Target for Age‐Related Disease,” Experimental & Molecular Medicine 55, no. 8 (2023): 1595–1619. 37612409 10.1038/s12276-023-01046-5 PMC10474116

[15] E. N. Gurzov and D. L. Eizirik , “Bcl‐2 Proteins in Diabetes: Mitochondrial Pathways of β‐Cell Death and Dysfunction,” Trends in Cell Biology 21, no. 7 (2011): 424–431. 21481590 10.1016/j.tcb.2011.03.001

[16] G. Amanakis and E. Murphy , “Cyclophilin D: An Integrator of Mitochondrial Function,” Frontiers in Physiology 11 (2020): 595. 32625108 10.3389/fphys.2020.00595 PMC7311779

[17] A. S. Rambold and J. Lippincott‐Schwartz , “Mechanisms of Mitochondria and Autophagy Crosstalk,” Cell Cycle 10, no. 23 (2011): 4032–4038. 22101267 10.4161/cc.10.23.18384 PMC3272286

[18] E. E. Elias , B. Lyons , and D. A. Muruve , “Gasdermins and Pyroptosis in the Kidney,” Nature Reviews. Nephrology 19, no. 5 (2023): 337–350. 36596918 10.1038/s41581-022-00662-0

[19] J. Zhou , J. Qiu , Y. Song , et al., “Pyroptosis and Degenerative Diseases of the Elderly,” Cell Death & Disease 14, no. 2 (2023): 94. 36755014 10.1038/s41419-023-05634-1 PMC9908978

[20] J. Ding , K. Wang , W. Liu , et al., “Pore‐Forming Activity and Structural Autoinhibition of the Gasdermin Family,” Nature 535, no. 7610 (2016): 111–116. 27281216 10.1038/nature18590

[21] R. Miao , C. Jiang , W. Y. Chang , et al., “Gasdermin D Permeabilization of Mitochondrial Inner and Outer Membranes Accelerates and Enhances Pyroptosis,” Immunity 56, no. 11 (2023): 2523–2541.e2528. 37924812 10.1016/j.immuni.2023.10.004 PMC10872579

[22] J. Yan , P. Wan , S. Choksi , and Z. G. Liu , “Necroptosis and Tumor Progression,” Trends Cancer 8, no. 1 (2022): 21–27. 34627742 10.1016/j.trecan.2021.09.003 PMC8702466

[23] K. Mizumura , S. M. Cloonan , K. Nakahira , et al., “Mitophagy‐Dependent Necroptosis Contributes to the Pathogenesis of COPD,” Journal of Clinical Investigation 124, no. 9 (2014): 3987–4003. 25083992 10.1172/JCI74985 PMC4151233

[24] C. G. Weindel , E. L. Martinez , X. Zhao , et al., “Mitochondrial ROS Promotes Susceptibility to Infection via Gasdermin D‐Mediated Necroptosis,” Cell 185, no. 17 (2022): 3214–3231.e3223. 35907404 10.1016/j.cell.2022.06.038 PMC9531054

[25] B. Gan , “Mitochondrial Regulation of Ferroptosis,” Journal of Cell Biology 220, no. 9 (2021): e202105043. 34328510 10.1083/jcb.202105043 PMC8329737

[26] D. Tang , X. Chen , and G. Kroemer , “Cuproptosis: A Copper‐Triggered Modality of Mitochondrial Cell Death,” Cell Research 32, no. 5 (2022): 417–418. 35354936 10.1038/s41422-022-00653-7 PMC9061796

[27] A. Petzold , R. Bowser , P. Calabresi , H. Zetterberg , and B. M. Uitdehaag , “Biomarker Time Out,” Multiple Sclerosis 20, no. 12 (2014): 1560–1563. 24557857 10.1177/1352458514524999

[28] X. Xie , R. Zhong , L. Luo , et al., “The Infection Characteristics and Autophagy Defect of Dermal Macrophages in STZ‐Induced Diabetic Rats Skin Wound *Staphylococcus aureus* Infection Model,” Immunity, Inflammation and Disease 9, no. 4 (2021): 1428–1438. 34647429 10.1002/iid3.492 PMC8589369

[29] X. Ji , P. Jin , P. Yu , and P. Wang , “Autophagy Ameliorates *Pseudomonas aeruginosa* ‐Infected Diabetic Wounds by Regulating the Toll‐Like Receptor 4/Myeloid Differentiation Factor 88 Pathway,” Wound Repair and Regeneration 31, no. 3 (2023): 305–320. 36879445 10.1111/wrr.13074

[30] I. Pastar , A. P. Sawaya , J. Marjanovic , et al., “Intracellular *Staphylococcus aureus* Triggers Pyroptosis and Contributes to Inhibition of Healing due to Perforin‐2 Suppression,” Journal of Clinical Investigation 131, no. 24 (2021): e133727. 34730110 10.1172/JCI133727 PMC8670843

[31] B. Yan , Y. Zhang , C. Liang , et al., “Stem Cell‐Derived Exosomes Prevent Pyroptosis and Repair Ischemic Muscle Injury Through a Novel Exosome/circHIPK3/FOXO3a Pathway,” Theranostics 10, no. 15 (2020): 6728–6742. 32550900 10.7150/thno.42259 PMC7295049

[32] H. Yang , Y. Zhang , Z. Du , T. Wu , and C. Yang , “Hair Follicle Mesenchymal Stem Cell Exosomal lncRNA H19 Inhibited NLRP3 Pyroptosis to Promote Diabetic Mouse Skin Wound Healing,” Aging (Albany NY) 15, no. 3 (2023): 791–809. 36787444 10.18632/aging.204513 PMC9970314

[33] S. Yang , M. Xu , G. Meng , and Y. Lu , “SIRT3 Deficiency Delays Diabetic Skin Wound Healing via Oxidative Stress and Necroptosis Enhancement,” Journal of Cellular and Molecular Medicine 24, no. 8 (2020): 4415–4427. 32119761 10.1111/jcmm.15100 PMC7176871

[34] J. T. Leek , W. E. Johnson , H. S. Parker , A. E. Jaffe , and J. D. Storey , “The Sva Package for Removing Batch Effects and Other Unwanted Variation in High‐Throughput Experiments,” Bioinformatics 28, no. 6 (2012): 882–883. 22257669 10.1093/bioinformatics/bts034 PMC3307112

[35] H. Qin , A. Abulaiti , A. Maimaiti , et al., “Integrated Machine Learning Survival Framework Develops a Prognostic Model Based on Inter‐Crosstalk Definition of Mitochondrial Function and Cell Death Patterns in a Large Multicenter Cohort for Lower‐Grade Glioma,” Journal of Translational Medicine 21, no. 1 (2023): 588. 37660060 10.1186/s12967-023-04468-x PMC10474752

[36] C. Peng , Y. Zhang , X. Lang , and Y. Zhang , “Role of Mitochondrial Metabolic Disorder and Immune Infiltration in Diabetic Cardiomyopathy: New Insights From Bioinformatics Analysis,” Journal of Translational Medicine 21, no. 1 (2023): 66. 36726122 10.1186/s12967-023-03928-8 PMC9893675

[37] M. E. Ritchie , B. Phipson , D. Wu , et al., “Limma Powers Differential Expression Analyses for RNA‐Sequencing and Microarray Studies,” Nucleic Acids Research 43, no. 7 (2015): e47. 25605792 10.1093/nar/gkv007 PMC4402510

[38] T. Wu , E. Hu , S. Xu , et al., “clusterProfiler 4.0: A Universal Enrichment Tool for Interpreting Omics Data,” Innovation (Cambridge) 2, no. 3 (2021): 100141. 10.1016/j.xinn.2021.100141 PMC8454663 34557778

[39] Y. Hao , S. Hao , E. Andersen‐Nissen , et al., “Integrated Analysis of Multimodal Single‐Cell Data,” Cell 184, no. 13 (2021): 3573–3587.e3529. 34062119 10.1016/j.cell.2021.04.048 PMC8238499

[40] Z. Wang , D. Wei , S. Li , et al., “Healing Mechanism of Diabetic Foot Ulcers Using Single‐Cell RNA‐Sequencing,” Annals of Translational Medicine 11, no. 5 (2023): 210. 37007553 10.21037/atm-23-240 PMC10061471

[41] G. Theocharidis , B. E. Thomas , D. Sarkar , et al., “Single Cell Transcriptomic Landscape of Diabetic Foot Ulcers,” Nature Communications 13, no. 1 (2022): 181. 10.1038/s41467-021-27801-8 PMC8748704 35013299

[42] S. Hänzelmann , R. Castelo , and J. Guinney , “GSVA: Gene Set Variation Analysis for Microarray and RNA‐Seq Data,” BMC Bioinformatics 14 (2013): 7. 23323831 10.1186/1471-2105-14-7 PMC3618321

[43] J. Alderden , G. A. Pepper , A. Wilson , et al., “Predicting Pressure Injury in Critical Care Patients: A Machine‐Learning Model,” American Journal of Critical Care 27, no. 6 (2018): 461–468. 30385537 10.4037/ajcc2018525 PMC6247790

[44] Y. Wang , B. Li , and Y. Zhao , “Inflammation in Preeclampsia: Genetic Biomarkers, Mechanisms, and Therapeutic Strategies,” Frontiers in Immunology 13 (2022): 883404. 35880174 10.3389/fimmu.2022.883404 PMC9307876

[45] J. Griss , G. Viteri , K. Sidiropoulos , V. Nguyen , A. Fabregat , and H. Hermjakob , “ReactomeGSA ‐ Efficient Multi‐Omics Comparative Pathway Analysis,” Molecular & Cellular Proteomics 19, no. 12 (2020): 2115–2125. 32907876 10.1074/mcp.TIR120.002155 PMC7710148

[46] S. Jin , C. F. Guerrero‐Juarez , L. Zhang , et al., “Inference and Analysis of Cell‐Cell Communication Using CellChat,” Nature Communications 12, no. 1 (2021): 1088. 10.1038/s41467-021-21246-9 PMC7889871 33597522

[47] P. Shannon , A. Markiel , O. Ozier , et al., “Cytoscape: A Software Environment for Integrated Models of Biomolecular Interaction Networks,” Genome Research 13, no. 11 (2003): 2498–2504. 14597658 10.1101/gr.1239303 PMC403769

[48] S. Lin , Z. Wen , S. Li , et al., “LncRNA Neat1 Promotes the Macrophage Inflammatory Response and Acts as a Therapeutic Target in Titanium Particle‐Induced Osteolysis,” Acta Biomaterialia 142 (2022): 345–360. 35151924 10.1016/j.actbio.2022.02.007

[49] A. Ashkenazi , W. J. Fairbrother , J. D. Leverson , and A. J. Souers , “From Basic Apoptosis Discoveries to Advanced Selective BCL‐2 Family Inhibitors,” Nature Reviews. Drug Discovery 16, no. 4 (2017): 273–284. 28209992 10.1038/nrd.2016.253

[50] A. R. Delbridge and A. Strasser , “The BCL‐2 Protein Family, BH3‐Mimetics and Cancer Therapy,” Cell Death and Differentiation 22, no. 7 (2015): 1071–1080. 25952548 10.1038/cdd.2015.50 PMC4572872

[51] F. J. Bock and S. W. G. Tait , “Mitochondria as Multifaceted Regulators of Cell Death,” Nature Reviews. Molecular Cell Biology 21, no. 2 (2020): 85–100. 31636403 10.1038/s41580-019-0173-8

[52] J. Chen , X. Li , H. Liu , et al., “Bone Marrow Stromal Cell‐Derived Exosomal Circular RNA Improves Diabetic Foot Ulcer Wound Healing by Activating the Nuclear Factor Erythroid 2‐Related Factor 2 Pathway and Inhibiting Ferroptosis,” Diabetic Medicine 40, no. 7 (2023): e15031. 36537855 10.1111/dme.15031

[53] J. Li , C. Jiang , and J. Xia , “The Role of Programmed Cell Death in Diabetic Foot Ulcers,” International Wound Journal 21, no. 2 (2023): e14399. 37736955 10.1111/iwj.14399 PMC10824602

[54] X. Wang , S. Dai , W. Zheng , et al., “Identification and Verification of Ferroptosis‐Related Genes in Diabetic Foot Using Bioinformatics Analysis,” International Wound Journal 20, no. 8 (2023): 3191–3203. 37249237 10.1111/iwj.14198 PMC10502281

[55] P. P. Ruvolo , X. Deng , and W. S. May , “Phosphorylation of Bcl2 and Regulation of Apoptosis,” Leukemia 15, no. 4 (2001): 515–522. 11368354 10.1038/sj.leu.2402090

[56] S. Pichu , S. Vimalraj , and V. Viswanathan , “Impact of microRNA‐210 on Wound Healing Among the Patients With Diabetic Foot Ulcer,” PLoS One 16, no. 7 (2021): e0254921. 34293021 10.1371/journal.pone.0254921 PMC8297780

[57] N. Zhang , H. Yu , T. Liu , et al., “Bmal1 Downregulation Leads to Diabetic Cardiomyopathy by Promoting Bcl2/IP3R‐Mediated Mitochondrial ca(2+) Overload,” Redox Biology 64 (2023): 102788. 37356134 10.1016/j.redox.2023.102788 PMC10320280

[58] X. Q. Liu , L. Jiang , Y. Y. Li , et al., “Wogonin Protects Glomerular Podocytes by Targeting Bcl‐2‐Mediated Autophagy and Apoptosis in Diabetic Kidney Disease,” Acta Pharmacologica Sinica 43, no. 1 (2022): 96–110. 34253875 10.1038/s41401-021-00721-5 PMC8724322

[59] M. Ni , A. Solmonson , C. Pan , et al., “Functional Assessment of Lipoyltransferase‐1 Deficiency in Cells, Mice, and Humans,” Cell Reports 27, no. 5 (2019): 1376–1386.e1376. 31042466 10.1016/j.celrep.2019.04.005 PMC7351313

[60] Y. Liu , G. Luo , Y. Yan , and J. Peng , “A Pan‐Cancer Analysis of Copper Homeostasis‐Related Gene Lipoyltransferase 1: Its Potential Biological Functions and Prognosis Values,” Frontiers in Genetics 13 (2022): 1038174. 36330439 10.3389/fgene.2022.1038174 PMC9623413

[61] Z. Lu , L. Ding , S. Zhang , et al., “Bioinformatics Analysis of Copper Death Gene in Diabetic Immune Infiltration,” Medicine (Baltimore) 102, no. 39 (2023): e35241. 37773841 10.1097/MD.0000000000035241 PMC10545334

[62] B. Chang , Z. Hu , L. Chen , Z. Jin , and Y. Yang , “Development and Validation of Cuproptosis‐Related Genes in Synovitis During Osteoarthritis Progress,” Frontiers in Immunology 14 (2023): 1090596. 36817415 10.3389/fimmu.2023.1090596 PMC9932029

[63] W. Wang , Z. Chen , and Y. Hua , “Bioinformatics Prediction and Experimental Validation Identify a Novel Cuproptosis‐Related Gene Signature in Human Synovial Inflammation During Osteoarthritis Progression,” Biomolecules 13, no. 1 (2023): 127. 36671512 10.3390/biom13010127 PMC9855951

[64] F. C. Song , J. Q. Yuan , M. D. Zhu , et al., “High Glucose Represses the Proliferation of Tendon Fibroblasts by Inhibiting Autophagy Activation in Tendon Injury,” Bioscience Reports 42, no. 3 (2022): BSR20210640. 35293974 10.1042/BSR20210640 PMC8935382

[65] L. Luo , Y. An , K. Geng , et al., “High Glucose‐Induced Endothelial STING Activation Inhibits Diabetic Wound Healing Through Impairment of Angiogenesis,” Biochemical and Biophysical Research Communications 668 (2023): 82–89. 37245293 10.1016/j.bbrc.2023.05.081

[66] Y. Liang , C. Yang , Y. Lin , et al., “Matrix Metalloproteinase 9 Induces Keratinocyte Apoptosis Through FasL/Fas Pathway in Diabetic Wound,” Apoptosis 24, no. 7–8 (2019): 542–551. 30949883 10.1007/s10495-019-01536-w

[67] C. C. Lan , C. S. Wu , S. M. Huang , I. H. Wu , and G. S. Chen , “High‐Glucose Environment Enhanced Oxidative Stress and Increased Interleukin‐8 Secretion From Keratinocytes: New Insights Into Impaired Diabetic Wound Healing,” Diabetes 62, no. 7 (2013): 2530–2538. 23423570 10.2337/db12-1714 PMC3712048

[68] L. Kuang , C. Zhang , B. Li , H. Deng , R. Chen , and G. Li , “Human Keratinocyte‐Derived Exosomal MALAT1 Promotes Diabetic Wound Healing by Upregulating MFGE8 via microRNA‐1914‐3p,” International Journal of Nanomedicine 18 (2023): 949–970. 36852184 10.2147/IJN.S399785 PMC9961177

[69] C.‐Y. Ko , W.‐C. Chang , and J.‐M. Wang , “Biological Roles of CCAAT/Enhancer‐Binding Protein Delta During Inflammation,” Journal of Biomedical Science 22, no. 1 (2015): 6. 25591788 10.1186/s12929-014-0110-2 PMC4318212

[70] K. Balamurugan and E. Sterneck , “The Many Faces of C/EBPδ and Their Relevance for Inflammation and Cancer,” International Journal of Biological Sciences 9, no. 9 (2013): 917–933. 24155666 10.7150/ijbs.7224 PMC3805898

[71] X. G. Mao , X. Y. Xue , R. Lv , et al., “CEBPD Is a Master Transcriptional Factor for Hypoxia Regulated Proteins in Glioblastoma and Augments Hypoxia Induced Invasion Through Extracellular Matrix‐Integrin Mediated EGFR/PI3K Pathway,” Cell Death & Disease 14, no. 4 (2023): 269. 37059730 10.1038/s41419-023-05788-y PMC10104878

[72] A. Forero , S. Ozarkar , H. Li , et al., “Differential Activation of the Transcription Factor IRF1 Underlies the Distinct Immune Responses Elicited by Type I and Type III Interferons,” Immunity 51, no. 3 (2019): 451–464.e456. 31471108 10.1016/j.immuni.2019.07.007 PMC7447158

[73] F. Geng , J. Chen , B. Song , et al., “Chaperone‐ and PTM‐Mediated Activation of IRF1 Tames Radiation‐Induced Cell Death and the Inflammatory Response,” Cellular & Molecular Immunology 21, no. 8 (2024): 856–872. 38849539 10.1038/s41423-024-01185-3 PMC11291999

[74] H. Feng , Y. B. Zhang , J. F. Gui , S. M. Lemon , and D. Yamane , “Interferon Regulatory Factor 1 (IRF1) and Anti‐Pathogen Innate Immune Responses,” PLoS Pathogens 17, no. 1 (2021): e1009220. 33476326 10.1371/journal.ppat.1009220 PMC7819612

