# Supplementary figures and images for: Bulk and Single‐Cell Transcriptome Analyses Unravel Gene Signatures of Mitochondria‐Associated Programmed Cell Death in Diabetic Foot Ulcer
Source: J Cell Mol Med. 2024 Dec 27;28(24):e70319. doi: 10.1111/jcmm.70319 (PMC11680196; doi:10.1111/jcmm.70319)

**A**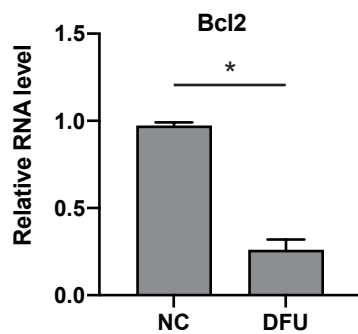**B**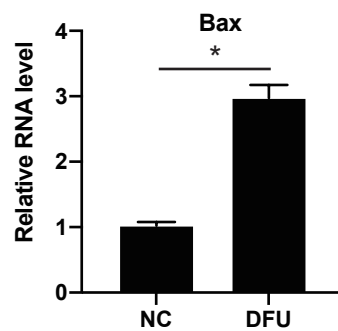**C**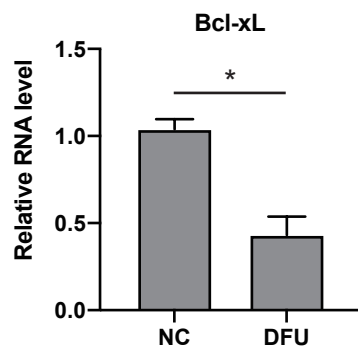**D**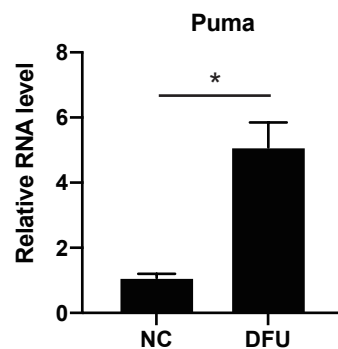

Supplement: Supplementary file 1 — Figure S1. The expression of BCL2‐related apoptosis genes in clinical tissue specimens. (A–D) PCR tests showed that Bcl‐2 and Bcl‐xL were downregulated and Bax and Puma were upregulated in the DFU group (p < 0.05). *p < 0.05. Two‐tailed Student’s t‐test was used for judging statistical significance with numerical results displayed in the form of mean ± SEM. All experiments were done in sextuplicate. [file JCMM-28-e70319-s003.pdf]
